# Learning from the Past–The Need for Empirical Evidence on the Transfer Effects of Computer Programming Skills

**DOI:** 10.3389/fpsyg.2016.01390

**Published:** 2016-09-14

**Authors:** Ronny Scherer

**Affiliations:** Faculty of Educational Sciences, Centre for Educational Measurement, University of OsloOslo, Norway

**Keywords:** computational thinking, computer programming, creativity, problem solving, transfer of learning

## Introduction to the problem

In recent years, education has put considerable emphasis on the development of twenty-first century skills—a set of skills that can almost universally be applied to a broad range of domains and problems, and that help students to deal with the challenges and demands of complex, real-world problem situations (Pellegrino and Hilton, 2012). Among others, these skills comprise problem solving, creativity, critical thinking, collaboration, adaptability, digital literacy, and computational thinking, and are considered to be critical in our information- and knowledge-rich society (Binkley et al., 2012; Wagner, 2012; Scherer, 2015; Care and Anderson, 2016). Against this background, it has become the designated aim of educators to help students to develop these skills (Kay and Greenhill, 2011). The question of *how* the development of these skills and the ability to transfer them to different contexts and knowledge domains can be fostered has therefore gained significance (Greiff et al., 2014). Nonetheless, this question is by no means trivial, because the transfer of knowledge and skills does not automatically happen, as Tricot and Sweller (2013) argued.

In the pursuit of finding ways to foster twenty-first century skills and their transfer, voices have become loud inspiring education to incorporate computer programming into K-12 curricula (Lye and Koh, 2014). The reactions on these voices have been tremendous; some countries developed an entire curriculum around computer programming (Sturman and Sizmur, 2011; Webb et al., 2016). Behind this development is the belief that fostering programming skills improves students' performance on other critical skills such as creativity and problem solving (Liao and Bright, 1991; Clements, 1995). Mitchel Resnick, the director of MIT's Media Lab and facilitator of the Scratch® programming language, argued that “programming supports “computational thinking,” helping you learn important problem-solving and design strategies […] that carry over to nonprogramming domains” (Resnick et al., 2009, p. 62). Along the same lines, Barr and Stephenson (2011) proposed that computer programming “is a problem solving methodology that can be automated and transferred and applied across subjects” (p. 51). Brown and Kölling (2012) took this argument even further and claimed that the “use of programming skills can allow for a deeper and more direct understanding of the subjects under investigation, using Computing to support learning in the same way that Mathematics supports the learning of subjects such as Physics.” (p. 1) Whereas there has been a great body of research supporting these claims in the 1980s and 1990s (for an overview, please refer to Liao and Bright, 1991), it seems as if there is very little evidence on the transfer effects of computer programming skills in the context of twenty-first century education (Grover and Pea, 2013; Lye and Koh, 2014). Although computer programming and other skills share a number of cognitive and even metacognitive processes (Clements, 1986, 1995; Brown and Kölling, 2012; Lye and Koh, 2014; Rich et al., 2014), therefore supporting potential transfer effects, I argue that educational research lags behind in sharing sufficient evidence for these claims.

Against this background, the main position this opinion paper conveys is that—although the conceptual argumentation about the potential transfer effects of computer programming skills on other skills such as problem solving and creativity is reasonable—there is a strong need for empirical evidence supporting this, particularly in the context of the recent advancements of digital technologies.

## Current status of knowledge

### Computer programming skills and the concept of computational thinking

Computer programming skills are considered to be an integral part of what is called “computational thinking” (CT; Denning, 2010; Lye and Koh, 2014), and often find their way into frameworks of digital literacy (Siddiq et al., 2016). CT has in fact gained importance in STEM education, and there is a growing interest in exploring how CT can be introduced in K-12 curricula (Lye and Koh, 2014). Wing (2006) defined CT as a thought process that “involves solving problems, designing systems, and understanding human behavior, by drawing on the concepts fundamental to computer science” (p. 33). In their review, Lye and Koh (2014) argued that computer programming—an activity that requires the abstraction and decomposition of problems—exposes students to these thinking processes, and claimed that it may therefore foster the development of CT. This claim has largely been supported by studies using the first (e.g., Logo®; Dyck and Mayer, 1989; Pardamean et al., 2015) and more recently developed programming tools for education (e.g., Scratch®; Wilson et al., 2013).

### Transfer effects on problem solving

Having established that computer programming and CT are closely connected, the question arises to what extent transfer effects on problem solving exist. The starting point for addressing this question is to examine which particular thinking processes are involved in programming and problem solving. According to Brooks (1999), Clements and Merriman (1988), and Denning (2010), programming comprises a number of processes that range from information retrieval and processes of understanding the problem at hand to discovering methods and algorithms that solve the problem, and evaluating them (Table 1). According to a framework proposed by the (OECD, 2014), domain-general problem solving comprises similar processes of exploring and understanding, representing and formulating, planning, and executing, and monitoring and reflecting (Table 1). These conceptualizations reveal a considerable overlap between the two constructs: The processes involved in programming are by and large of the same nature as those involved in problem solving, in that the problem space needs to be understood and explored first, hypotheses and methods are to be developed second, and the proposed solution—be it an algorithm, product, or any other problem solution—finally needs to be evaluated (Klahr and Dunbar, 1988). This parallelism brings forth the question whether computer programming can actually be considered a form of problem solving (Barr and Stephenson, 2011; Jonassen, 2011), and suggests that transfer effects may exist. Investigating these ideas systematically, Liao and Bright (1991) conducted a meta-analysis of 65 studies that were conducted between 1969 and 1989 with the goal to synthesize empirical evidence on the effects of computer programming on problem solving abilities. The authors found an average effect size of 41 and concluded that students—while learning to program—acquire reasoning, logical thinking, and planning (i.e., problem solving) skills that go beyond computer programming. This meta-analysis was, however, followed by only a very limited number of experimental studies that continued examining these effects (Maloney et al., 2004; Gibbon, 2007; Pardamean et al., 2015), some of which were insignificant (Lai and Yang, 2011; Gülbahar and Kalelioğlo, 2014; Korkmaz, 2016). This is somehow surprising, because Liao and Bright (1991) clearly showed that the average effect was moderated by the type of programming environment used in the treatment group, thus suggesting that further advancements in these tools may affect the transfer effect. In fact, many publications that argued for the transfer effects later on only described programming tools or feasibility studies thereof on a conceptual level (Grover and Pea, 2013). By contrast, a larger body of research exists on the transfer effects on mathematical thinking and conceptual understanding (e.g., Calder, 2010; Kazakoff et al., 2013; Rich et al., 2014).

**Table 1:** **Key processes involved in computer programming, problem solving, and creative thinking**.

**Computer programming**	**Problem solving**	**Creative thinking**
▪ Representing, storing, and retrieving information in order to understand the problem (i.e., knowledge acquisition of basic problem elements such as objects, relations, initial and final states of objects) ▪ Discovering algorithms for information processes —finding a method in order to represent the real-world problem, develop, and execute an action plan and code ▪ Evaluating the performance of the designed complex systems on the basis of the written code; bridging the gap between the problem statement and the solution	▪ Exploring and understanding the problem (e.g., by decomposing the problem into sub-problems) ▪ Representing and formulating the problem by creating representations of the problem situation and formulating hypotheses ▪ Planning and executing the sequential steps to solve the problem ▪ Monitoring progress and reflecting on the problem, the solution, and solution strategy	▪ Acquiring knowledge and skills relevant to the creative act, setting goals; encoding, recognizing, and formulating the problem (preparation) ▪ Building a representation of the problem (e.g., by combining components of the problem) and unconscious processing (incubation) ▪ Searching for and finding solutions (illumination) ▪ Evaluating the creative product, monitoring the process of creative activities, and improving shortcomings (verification)

### Transfer effects on creativity

Similar to the reasoning on the transfer effects on problem solving, researchers have claimed that learning to program fosters students' creative thinking. In the most recent systematic review that I could identify (Clements, 1995), the author found that computer programming instruction fosters creativity and divergent thinking; significant effects on originality—a facet of creative thinking that involves selective encoding and combining—could be identified. Clements (1995) concluded his review by summarizing the key elements of computer programming that are also essential for creative thinking: decide on the nature of the problem, combine components of the problem, select a mental representation, monitor progress, acquire knowledge, and encode (see also Clements and Merriman, 1988). In a slightly different framework, Wallas (1926) proposed four stages of creativity, namely preparation, incubation, illumination, and verification, which are still widely used today. Comparing these processes with those required for computer programming (Table 1), substantive similarities exist, such that transfer effects between them could be expected. In fact, programming is considered to be a creative human activity (Denning, 2010; Grover and Pea, 2013). Yet, the existing body of empirical evidence supporting this expectation is rather meager: Except for a single study (Pardamean, 2014), the bulk of published empirical research on the transfer effects on creativity dates back to the 1980s and 1990s (Clements, 1986, 1991, 1995; McGrath, 1988; Subhi, 1999).

## What is needed

In light of this brief review of the current status of knowledge, I would like to follow Mayer (2015), who discussed the strong need for research evidence on game-based learning, and propose that research on the transfer effects of computer programming needs to move beyond untested claims and the mere description of programming tools, their feasibility, and students' interest or enjoyment thereof Fessakis et al. (2013) raised similar concerns: “Instead of focussing on the cognitive effects of programming, more recent studies concern the development of new programming environments for children […]” (p. 89) Along these lines, educational research needs to focus more on the cognitive consequences of learning to program by designing experimental studies that systematically evaluate the transfer effects, making use of the well-developed programming tools such as Scratch® (Resnick et al., 2009). I believe that the outstanding number of feasibility studies on these tools create a fruitful ground for experimental comparisons. Such comparisons, however, need an appropriate research design, which fulfills at least three criteria (Mayer, 2015): (1) Appropriate *outcome measures* of academic learning or other skills that go beyond students' self-reports, enjoyment, or interest; (2) *Experimental control*—pretest-posttest designs with a treatment group (i.e., the group that learns to program) and a control group; (3) *Random assignment* of students to treatment and control groups. Despite the very few studies during the last 20 years, which adhered to these criteria (e.g., Pardamean et al., 2015), my observation is that research on the transfer effects of computer programming is in need of methodologically sound, experimental studies.

## Conclusion

From a conceptual perspective, the claims that learning computer programming may translate into the development of other, cognate skills such as problem solving or creative thinking, do have their standing, particularly because a considerable conceptual overlap between these skills exists. From an empirical perspective though, it seems as if the conceptual claims have been supplemented with evidence from experimental studies to a very limited extent. My observation is that most of the empirical research on the transfer effects dates back to the 1980s and 1990s; yet, too few studies have looked into these effects in the twenty-first century. This observation is somehow unexpected, particularly because Pea and Kurland (1984) pointed to the strong need for evidence on the transfer effects of computer programming that takes into account the development of digital technologies more than 20 years ago. Although this plea has been followed by a wave of experimental studies (Liao and Bright, 1991; Clements, 1995), educational research has not systematically followed up on examining the transfer effects. On the basis of the limited research on the transfer effects of computer programming skills on other cognitive skills on the one hand, and the conceptual claims that these transfer effects exist on the other hand, I would like to encourage researchers to fill this gap by learning from the past and reviving this research area in the twenty-first century.

## Author contribution

RS has conducted the literature review, drafted the manuscript, and performed revisions.

### Conflict of interest statement

The author declares that the research was conducted in the absence of any commercial or financial relationships that could be construed as a potential conflict of interest.
